# Testing Association between Mixed Type Outcomes and Covariates Jointly by the Use of a Latent Variable

**DOI:** 10.1038/s41598-017-08371-6

**Published:** 2017-08-14

**Authors:** Jiayan Zhu, Wei Zhang, Qizhai Li, Zhengbang Li

**Affiliations:** 1 School of Mathematics and Statistics & Hubei Key Laboratory of Mathematical Sciences, Central China Normal University, Wuhan, 430079 China; 2 School of Information and Communication, Wuhan College, Wuhan, 430212 China; 3 Academy of Mathematics and Systems Science, Chinese Academy of Sciences, Beijing, 100190 China

## Abstract

Multiple outcomes are often collected simultaneously in biomedical fields in order to identify whether a continuous response and an ordinal response are associated with some covariates simultaneously. Here we propose a joint statistical model by the use of a latent variable underlying the ordinal response. Asymptotic results are obtained and a jointly test is proposed for testing the continuous response and the ordinal response are associated with some covariates simultaneously. Extensive simulations and real data analysis results indicate more efficient performances of the proposed method than that of the combined p-values method.

## Introduction

Multiple outcomes are often encountered in a variety of fields including social sciences, economics, and biomedical, in order to characterize the effect of a covariate or investigate the association between multiple outcomes and the interested variables. In many cases, these outcomes are of mixed types in the sense that some are continuous, and others may be ordinal. For example, in a mental health study^1^ in Florida, USA, the collected outcomes consist of mental impairment, life events, and socioeconomic status(SES) which are of different data types. The mental impairment is ordinal, with 4 categories(1 = well, 2 = mild symptom, 3 = moderate symptom formation, 4 = impaired). For the life events index, it is a composite measure which includes the number and severity of important life events occurred to the subject within the past 3 years, such as the birth of a child, a new job, a divorce, or a death in the family. The life events index can be taken as a continuous response. SES is measured as binary which can be taken as a covariate. Investigators want to judge whether mental impairment or life events index is associated with SES.

Various approaches have been developed to model mixed outcomes in the literature. A direct procedure is to ignore the correlations among multiple outcomes and fit each outcome with a model that best suits its type separately. Dale^2^ proposed global cross-ratio models as a measure of association for bivariate, discrete and ordinal responses. For testing association between a bivariate trait with a continuous and discrete (taking values 0, 1) outcomes and a covariate jointly, a widely used class of approaches are latent variable models. Catalano and Ryan^3^ proposed a bivariate latent variable models for clustered discrete and continuous outcomes with the joint distirbution being a product of a standard random effects model for the continuous variable and a probit model for the discrete variable. Fitzmaurice and Laird^4^ proposed a model for a correlated binary outcome and a continuous outcome based on the factorization of the joint distribution of the outcomes. Sammel *et al*.^5^ presented a latent variable model by assuming that the observed outcomes are physical manifestations of a latent variable. Gueorguieva *et al*.^6^ proposed a correlated probit model to model clustered binary and continuous responses jointly. Teixeira-Pinto *et al*.^7^ provided a new joint model for a binary and a continuous outcomes. In genome-wide association analysis field, Liu *et al*.^8^ developed an extended generalized estimating equation method for bivariate association analyses of continuous and binary traits. Their simulation results demonstrated that, compared with univariate analysis, bivariate analysis could substantially improve power while having comparable type I error rates under certain situations. Yuan *et al*.^9^ extended the joint linkage analysis of multivariate qualitative and quantitative traits described by Williams *et al*.^10, 11^ to association analysis. Yuan *et al*.^9^ also assumed two latent variables specified for the qualitative and quantitative traits followed a bivariate normal distribution. With such modeling, likelihood-based inference procedures are introduced to test for pleiotropic genetic effects.

In dealing with an single ordinal(taking values 1, 2, …) outcome, the proportional odds model^12^ is usually adopted. In particular, the proportional odds model with the outcome belonging to a set of ordered categories can be regarded as an extension of the logistic regression model for binary outcomes. It can be expressed as a series of logistic regression models for dependent binary variables with common regression parameters reflecting the proportional odds assumption^13^. When the outcome is continuous, the ordinary linear model is the most often used. In order to test the association between a bivariate trait with a continuous and ordinal outcomes and some covariates jointly, we also applied a latent variable model and proposed a statistical approach to model the ordinal outcome and continuous outcome simultaneously. Let us begin with two ordinary linear models *Y*_1_ = *α*_0_ + *G* ^*τ*^ *α* + *ε*_1_, and *Z* = *β*_0_ + *G* ^*τ*^ *β* + *ε*_2_ where *Y*_1_ is a continuous response, *Z* is a latent continuous variable, *G* = (*G* ^(1)^, $\cdots$, *G* ^(*p*)^)^*τ*^ represents a *p*-dimensional covariate, *α*_0_ and *β*_0_ are the intercept parameters, *β* = (*β*_1_, $\cdots$, *β*_*p*_)^*τ*^ and *α* = (*α*_1_, $\cdots$, *α*_*p*_)^*τ*^ are parameters, and (*ε*_1_,*ε*_2_)^*τ*^ follow the bivariate normal distribution with mean vector (0, 0)^*τ*^ and symmetric covariance matrix consisting of diagonal elements being ${\sigma }_{1}^{2}$, and ${\sigma }_{2}^{2}$, and **off-diagonal** elements both being *ρσ*_1_ *σ*_2_, respectively. We note that standard errors *σ*_1_, *σ*_2_ and correlation coefficient *ρ* are unknown. In addition, suppose that *Z* is a latent variable underlied an observed ordinal response *Y*_2_ which can be observed in the following manners: *Y*_2_ = 1 as −∞ < *Z* ≤ *γ*_1_; *Y*_2_ = 2 as *γ*_1_ < *Z* ≤ *γ*_2_; $\cdots$; *Y*_2_ = *k* as *γ*_*k*−1_ < *Z* ≤ *γ*_*k*_; *Y*_2_ = *k* + 1 as *γ*_*k*_ < *Z* ≤ ∞, where *k* is an integer related to the number of categories for *Y*_2_ and *γ*_1_,*γ*_2_, $\cdots$, *γ*_*k*_ are *k* ordered cutpoint values. Note that the latent variable *Z* is unobserved.

Suppose *n* independent observations $({Y}_{1i},{Y}_{2i},{G}_{i}^{\tau })$ are available for (*Y*_1_, *Y*_2_, *G* ^*τ*^), where *G*_*i*_ = (*g*_1*i*_, $\cdots$, *g*_*pi*_)^*τ*^, *i* = 1, $\cdots$, *n*. Based on the foregoing assumption, (*Y*_1*i*_, *Z*_*i*_)^*τ*^ are independently distributed **as** a bivariate normal distribution with mean vector ${({\alpha }_{0}+{G}_{i}^{\tau }\alpha ,{\beta }_{0}+{G}_{i}^{\tau }\beta )}^{\tau }$. The conditional distribution of *Z*_*i*_ given *Y*_1*i*_ is normal with mean $({\beta }_{0}+{G}_{i}^{\tau }\beta )+({Y}_{1i}-{G}_{i}^{\tau }\alpha -{\alpha }_{0})\rho \frac{{\sigma }_{2}}{{\sigma }_{1}}$ and variance ${\sigma }_{2}^{2}\mathrm{(1}-{\rho }^{2})$. By some algebra, for *j* = 1, $\cdots$, *k*, and *i* = 1, …, *n*, it can be obtained that

1 $$\begin{array}{rcl}{\rm{\Pr }}({Z}_{i}\le {\gamma }_{j}|{Y}_{1i}) & = & Pr\{\frac{{Z}_{i}-[({\beta }_{0}+{G}_{i}^{\tau }\beta )+({Y}_{1i}-{\alpha }_{0}-{G}_{i}^{\tau }\alpha )\rho \frac{{\sigma }_{2}}{{\sigma }_{1}}]}{\sqrt{{\sigma }_{2}^{2}\mathrm{(1}-{\rho }^{2})}}\le \frac{{\gamma }_{j}-[({\beta }_{0}+{G}_{i}^{\tau }\beta )+({Y}_{1i}-{\alpha }_{0}-{G}_{i}^{\tau }\alpha )\rho \frac{{\sigma }_{2}}{{\sigma }_{1}}]}{\sqrt{{\sigma }_{2}^{2}\mathrm{(1}-{\rho }^{2})}}|{Y}_{1i}\}\\  & = & {\rm{\Phi }}\{\frac{{\gamma }_{j}-[({\beta }_{0}+{G}_{i}^{\tau }\beta )+({Y}_{1i}-{\alpha }_{0}-{G}_{i}^{\tau }\alpha )\rho \frac{{\sigma }_{2}}{{\sigma }_{1}}]}{\sqrt{{\sigma }_{2}^{2}\mathrm{(1}-{\rho }^{2})}}\}\\  & = & {\rm{\Phi }}[\frac{{\gamma }_{j}}{{\sigma }_{2}\sqrt{1-{\rho }^{2}}}-(\frac{{\beta }_{0}}{{\sigma }_{2}\sqrt{1-{\rho }^{2}}}-\frac{{\alpha }_{0}\rho }{{\sigma }_{1}\sqrt{1-{\rho }^{2}}})-{G}_{i}^{\tau }(\frac{\beta }{{\sigma }_{2}\sqrt{1-{\rho }^{2}}}-\frac{\alpha \rho }{{\sigma }_{1}\sqrt{1-{\rho }^{2}}})-{Y}_{1i}\frac{\rho }{{\sigma }_{1}\sqrt{1-{\rho }^{2}}}],\end{array}$$

where, Φ(·) is the standard normal distribution function.

Denote ${\beta }_{0}^{\ast }=\frac{{\beta }_{0}}{{\sigma }_{2}\sqrt{1-{\rho }^{2}}}$, ${\beta }^{\ast }=\frac{\beta }{{\sigma }_{2}\sqrt{1-{\rho }^{2}}}$, ${\gamma }_{j}^{\ast }=\frac{{\gamma }_{j}}{{\sigma }_{2}\sqrt{1-{\rho }^{2}}}$, and ${\theta }^{\ast }=\frac{\rho }{{\sigma }_{1}\sqrt{1-{\rho }^{2}}}$. We have

2 $${{\rm{\Phi }}}^{-1}[Pr({Z}_{i}\le {\gamma }_{j}|{Y}_{1i})]={\gamma }_{j}^{\ast }-{\beta }_{0}^{\ast }-{G}_{i}^{\tau }{\beta }^{\ast }-({Y}_{1i}-{\alpha }_{0}-{G}_{i}^{\tau }\alpha ){\theta }^{\ast },$$

for *j* = 1, $$\cdots $$, *k*, and *i* = 1, …, *n*.

In this paper, we propose a joint test for testing the association between a bivariate with an ordinal response and a continuous response and the covariates of interest. We derive the asymptotic properties for the estimators for parameters of interested covariates in the joint model. Extensive simulations are conducted to compare the performances of our proposed method to those of the existing combined p-values method. Application to the aforementioned mental impairment study in Florida further demonstrate good performances of our new method.

## Results

### Joint Model for a Bivariate with a continuous response and an ordinal responses and a Covariate

For $i=1,\cdots ,n$, ${Y}_{1i}-{\alpha }_{0}-{G}_{i}^{\tau }\alpha$ represents error term and has expectation value 0, so *β* ^*^ in (2) can be taken as a measure for the association between the ordinal response *Y*_2_ and covariates *G* after adjusting the effect of continuous response *Y*_1_. But ${Y}_{1i}-{\alpha }_{0}-{G}_{i}^{\tau }\alpha$ is unobserved for $i=1,\cdots ,n.$ Alternatively, a more common procedure instead of (2) is to use

3 $${{\rm{\Phi }}}^{-1}[{\rm{\Pr }}({Z}_{i}\le {\gamma }_{j}|{Y}_{1i})]={\gamma }_{j}^{\ast }-{\beta }_{0}^{\ast \ast }-{G}_{i}^{\tau }{\beta }^{\ast \ast }-{Y}_{1i}{\theta }^{\ast },$$

where *β* ^**^ = *β* ^*^ − *α* ^*^, ${\beta }_{0}^{\ast \ast }={\beta }_{0}^{\ast }-{\alpha }_{0}^{\ast }$, *α* ^*^ = *αθ* ^*^, and ${\alpha }_{0}^{\ast }={\alpha }_{0}{\theta }^{\ast }$. It is obvious that the model (3) links the ordinal response *Y*_2_ with *G* and *Y*_2_ with a similar manner as the proportional odds model. The main difference is that (3) uses the standard normal distribution Φ as the link function, while logit function is utilized by the proportional odds model.

With *α* = *α* ^*^/*θ* ^*^, the joint model of continuous variable *Y*_1_ and ordinal variable *Y*_2_ can be constructed as follows:

as *j* = 1,

4 $$\{\begin{array}{rcl}{\rm{\Pr }}({Y}_{1} < {y}_{1}) & = & {\int }_{-\infty }^{{y}_{1}}\frac{1}{\sqrt{2\pi {\sigma }_{1}^{2}}}\exp [\frac{-{(v-\frac{{\alpha }_{0}^{\ast }}{{\theta }^{\ast }}-{G}^{\tau }\frac{{\alpha }^{\ast }}{{\theta }^{\ast }})}^{2}}{2{\sigma }_{1}^{2}}]dv\\ {\rm{\Pr }}({Y}_{2}=j|{Y}_{1}) & = & {\rm{\Phi }}({\gamma }_{1}^{\ast }-{\beta }_{0}^{\ast \ast }-{G}^{\tau }{\beta }^{\ast \ast }-{Y}_{1}{\theta }^{\ast })\end{array};$$

as *j* = 2, $$\cdots $$, *k*,

5 $$\{\begin{array}{rcl}{\rm{\Pr }}({Y}_{1} < {y}_{1}) & = & {\int }_{-\infty }^{{y}_{1}}\frac{1}{\sqrt{2\pi {\sigma }_{1}^{2}}}\exp [\frac{-{(v-\frac{{\alpha }_{0}^{\ast }}{{\theta }^{\ast }}-{G}_{i}^{\tau }\frac{{\alpha }^{\ast }}{{\theta }^{\ast }})}^{2}}{2{\sigma }_{1}^{2}}]dv\\ {\rm{\Pr }}({Y}_{2}=j|{Y}_{1}) & = & {\rm{\Phi }}({\gamma }_{j}^{\ast }-{\beta }_{0}^{\ast \ast }-{G}^{\tau }{\beta }^{\ast \ast }-{Y}_{1}{\theta }^{\ast })-{\rm{\Phi }}({\gamma }_{j-1}^{\ast }-{\beta }_{0}^{\ast \ast }-{G}^{\tau }{\beta }^{\ast \ast }-{Y}_{1}{\theta }^{\ast })\end{array};$$

as *j* = *k* + 1,

6 $$\{\begin{array}{rcl}{\rm{\Pr }}({Y}_{1} < {y}_{1}) & = & {\int }_{-\infty }^{{y}_{1}}\frac{1}{\sqrt{2\pi {\sigma }_{1}^{2}}}{\rm{e}}{\rm{x}}{\rm{p}}[\frac{-{(v-\frac{{\alpha }_{0}^{\ast }}{{\theta }^{\ast }}-{G}_{i}^{\tau }\frac{{\alpha }^{\ast }}{{\theta }^{\ast }})}^{2}}{2{\sigma }_{1}^{2}}]dv\\ {\rm{\Pr }}({Y}_{2}=j|{Y}_{1}) & = & 1-{\rm{\Phi }}({\gamma }_{k}^{\ast }-{\beta }_{0}^{\ast \ast }-{G}_{i}^{\tau }{\beta }^{\ast \ast }-{Y}_{1}{\theta }^{\ast })\end{array}.$$

### Testing Association between a Bivariate and a Covariate

The maximum likelihood estimates (MLEs) for parameters ${\alpha }_{0}^{\ast }$, ${\beta }_{0}^{\ast \ast }$, *α* ^*^, *β* ^**^, *θ* ^*^, ${\gamma }_{1}^{\ast },\cdots ,{\gamma }_{k}^{\ast }$, and ${\sigma }_{1}^{2}$ can be obtained by maximizing the log-likelihood function which is implemented by solving score equations in the method section. Denote $a={({\alpha }_{0}^{\ast },{\beta }_{0}^{\ast \ast },{\alpha }^{\ast },{\beta }^{\ast \ast },{\theta }^{\ast },{\gamma }_{1}^{\ast },\cdots ,{\gamma }_{k}^{\ast },{\sigma }_{1}^{2})}^{\tau }$ and denote the corresponding MLE by $\hat{a}={(\widehat{{\alpha }_{0}^{\ast }},\widehat{{\beta }_{0}^{\ast \ast }},\widehat{{\alpha }^{\ast }},\widehat{{\beta }^{\ast \ast }},\widehat{{\theta }^{\ast }},\widehat{{\gamma }_{1}^{\ast }},\cdots ,\widehat{{\gamma }_{k}^{\ast }},\widehat{{\sigma }_{1}^{2}})}^{\tau }$. Following the statistical asymptotical theory^14^, under the null hypothesis where neither the continuous response *Y*_1_ nor the ordinal response *Y*_2_ is associated with covariates *G*, we have $\sqrt{n}({\hat{a}}^{\tau }-{a}^{\tau })$ asymptotically follows from the normal distribution with mean vector 0_(2*p*+*k*+4)×1_ and covariance *I* ^−1^(*a*), where *I*(*a*) is the Fisher information matrix(see method section). As mentioned earlier, *β* ^*^ in (2) measure the association between the ordinal response *Y*_2_ and *G* by adjusting the effect of continuous response *Y*_1_. Denote $\widehat{{\beta }^{\ast }}=\widehat{{\beta }^{\ast \ast }}+\widehat{{\alpha }^{\ast }}$ and let *V* be a submatrix corresponding to the 3rd to the (2*p* + 2)th rows and columns of *I* ^−1^(*a*), and *B* = (*I*_*p*_, *I*_*p*_), where *I*_*p*_ is a *p* × *p* identity matrix. Then we can conclude that $\widehat{{\beta }^{\ast }}$ is an asymptotic unbiased estimate for the parameter *β* ^*^ and it follows asymptotically from the normal distribution with mean vector *β* ^**^ + *α* ^*^ and covariance matrix *BVB* ^*τ*^ as the sample size *n* goes to infinity according to the multi-normal distribution theory^15^.

Our goal of this paper is to test whether the bivariate with the outcomes of mixed types (*Y*_1_ and *Y*_2_) is associated with covariates *G* of interest. The null hypothesis is neither the continuous response *Y*_1_ nor the ordinal response *Y*_2_ is associated with the variable *G*. Denote $\widehat{\eta }={(\widehat{{\alpha }_{1}^{\ast }},\cdots ,\widehat{{\alpha }_{p}^{\ast }},\widehat{{\beta }_{1}^{\ast }},\cdots ,\widehat{{\beta }_{p}^{\ast }})}^{\tau }$ and $W={\rm{var}}(\widehat{\eta })$. We can obtain *W* = *HVH* ^*τ*^, where, $$H={(\begin{array}{c}{I}_{p}{\mathrm{,0}}_{p}\\ {I}_{p},{I}_{p}\end{array})}_{2p\times 2p}$$. The detailed derivation of *W* is displayed in method section. With this, we propose a joint test denoted by

7 $${\rm{JT}}={(\widehat{\eta })}^{\tau }{(\widehat{W})}^{-1}\widehat{\eta },$$

which follows from a Chi-Squared distribution with degree freedom of 2*p* under the null hypothesis, where $\widehat{W}=H\widehat{V}{H}^{\tau }$ is a consistent estimate of the covariance matrix *W*, and $\widehat{V}$ is a consistent estimate of the covariance matrix *V* corresponding to the 3rd to the 2*p* + 2th rows and columns of ${I}^{-1}(\hat{a})$.

### Simulation Results

In this section, we explore the performances of JT by comparing it to a method of combined p-values^16^ (denoted by CP). In our case, CP can be implemented as: firstly, we apply the ordinary linear model to regressing *Y*_1_ on *G* and calculate the p-value denoted by pv_1_ for testing the association between *Y*_1_ and *G*; secondly, we apply the proportional odd model to regressing the ordinal response *Y*_2_ on *G* and denote the resulted p-value by pv_2_; lastly, we use the statistic CP = −2log(*pv*_1_) − 2log(*pv*_2_) as the final test. The p-value of CP can be calculated by the permutation method with 200 iterations. Assume that *p* = 1, and *n* = 500. We generate a 1-dimensional covariate *G* from standard normal distribution with sample size *n* and fix it as a covariate in our simulation iterations. The correlated error terms can be sampled from a bivariate normal distribution with mean vector (0, 0)^*τ*^ and covariance matrix in which its diagonal elements are both equal to 1 and the non-diagonal element is equal to *ρ*. We generate continuous variable *Y*_1_ and the latent continuous variable *Z* based on the former two linear models in introduction section with parameter *α*_0_, *β*_0_, *α*_1_ and *β*_1_. We consider 2 scenarios with 3 ordinal category values(*k* = 2) and 4 ordinal category values(*k* = 3). Tables 1 and 2 show the empirical type I error rates for *k* = 2 and *k* = 3 respectively. Tables 3 and 4 show the empirical powers for *k* = 2 and *k* = 3 respectively.

**Table 1:** Empirical type-1 error rates for tests JT and CP with 3 ordinal categories(*k* = 2).

*ρ*	*α*_1_	*β*_1_	JT	CP	*ρ*	*α*_1_	*β*_1_	JT	CP
*α*_0_ = 0.2, *β*_0_ = 0.2									
0.3	0	0	0.047	0.052	0.7	0	0	0.048	0.049
0.4	0	0	0.052	0.048	0.8	0	0	0.050	0.046
0.5	0	0	0.048	0.051	0.9	0	0	0.047	0.050
0.6	0	0	0.049	0.047	0.99	0	0	0.049	0.053
*α*_0_ = 1.2, *β*_0_ = 1.2									
0.3	0	0	0.046	0.049	0.7	0	0	0.052	0.049
0.4	0	0	0.053	0.050	0.8	0	0	0.047	0.048
0.5	0	0	0.048	0.048	0.9	0	0	0.048	0.053
0.6	0	0	0.051	0.052	0.99	0	0	0.049	0.051

**Table 2:** Empirical type-1 error rates for tests JT and CP with 4 ordinal categories(*k* = 3).

*ρ*	*α*_1_	*β*_1_	JT	CP	*ρ*	*α*_1_	*β*_1_	JT	CP
*α*_0_ = 0.2, *β*_0_ = 0.2									
0.3	0	0	0.048	0.054	0.7	0	0	0.049	0.045
0.4	0	0	0.050	0.046	0.8	0	0	0.052	0.048
0.5	0	0	0.046	0.049	0.9	0	0	0.048	0.051
0.6	0	0	0.051	0.052	0.99	0	0	0.050	0.053
*α*_0_ = 1.2, *β*_0_ = 1.2									
0.3	0	0	0.049	0.050	0.7	0	0	0.046	0.051
0.4	0	0	0.047	0.046	0.8	0	0	0.46	0.049
0.5	0	0	0.052	0.045	0.9	0	0	0.049	0.048
0.6	0	0	0.050	0.050	0.99	0	0	0.048	0.053

**Table 3:** Empirical powers for tests JT and CP with 3 ordinal categories(*k* = 2).

*ρ*	*α*_1_	*β*_1_	JT	CP	*ρ*	*α*_1_	*β*_1_	JT	CP
*α*_0_ = 0.2, *β*_0_ = 0.2									
0.3	0	0.15	0.802	0.748	0.7	0	0.15	0.882	0.588
0.3	0.05	0.15	0.748	0.732	0.7	0.05	0.15	0.756	0.582
0.3	0.15	0.15	0.922	0.948	0.7	0.15	0.15	0.85	0.894
0.3	0.15	0.05	0.832	0.86	0.7	0.15	0.05	0.868	0.702
0.3	0.15	0	0.8	0.792	0.7	0.15	0	0.964	0.744
0.4	0	0.15	0.782	0.64	0.8	0	0.15	0.966	0.586
0.4	0.05	0.15	0.752	0.746	0.8	0.05	0.15	0.86	0.626
0.4	0.15	0.15	0.908	0.942	0.8	0.15	0.15	0.84	0.886
0.4	0.15	0.05	0.822	0.802	0.8	0.15	0.05	0.946	0.69
0.4	0.15	0	0.846	0.792	0.8	0.15	0.05	0.99	0.716
0.5	0	0.15	0.802	0.644	0.9	0	0.15	0.998	0.558
0.5	0.05	0.15	0.724	0.676	0.9	0.05	0.15	0.922	0.57
0.5	0.15	0.15	0.906	0.932	0.9	0.15	0.15	0.832	0.866
0.5	0.15	0.05	0.84	0.784	0.9	0.15	0.05	0.98	0.674
0.5	0.15	0	0.896	0.780	0.9	0.15	0	0.982	0.646
0.6	0	0.15	0.852	0.618	0.99	0.05	0.15	0.986	0.622
0.6	0.05	0.15	0.778	0.686	0.99	0.05	0.15	0.98	0.54
0.6	0.15	0.15	0.886	0.904	0.99	0.15	0.15	0.77	0.836
0.6	0.15	0.05	0.862	0.77	0.99	0.15	0.05	0.99	0.606
0.6	0.15	0	0.922	0.768	0.99	0.15	0.05	0.996	0.774
*α*_0_ = 1.2, *β*_0_ = 1.2									
0.3	0	0.15	0.758	0.684	0.7	0	0.15	0.924	0.622
0.3	0.05	0.15	0.72	0.682	0.7	0.05	0.15	0.842	0.700
0.3	0.15	0.15	0.912	0.942	0.7	0.15	0.15	0.952	0.958
0.3	0.15	0.05	0.766	0.76	0.7	0.15	0.05	0.948	0.844
0.3	0.15	0	0.77	0.768	0.7	0.15	0	0.976	0.846
0.4	0	0.15	0.708	0.588	0.8	0	0.15	0.94	0.512
0.4	0.05	0.15	0.71	0.668	0.8	0.05	0.15	0.876	0.674
0.4	0.15	0.15	0.89	0.918	0.8	0.15	0.15	0.938	0.948
0.4	0.15	0.05	0.76	0.74	0.8	0.15	0.05	0.964	0.812
0.4	0.15	0	0.838	0.758	0.8	0.15	0.05	0.982	0.682
0.5	0	0.15	0.784	0.598	0.9	0	0.15	0.998	0.47
0.5	0.05	0.15	0.716	0.658	0.9	0.05	0.15	0.916	0.594
0.5	0.15	0.15	0.87	0.92	0.9	0.15	0.15	0.924	0.942
0.5	0.15	0.05	0.79	0.724	0.9	0.15	0.05	0.986	0.788
0.5	0.15	0	0.88	0.782	0.9	0.15	0	0.994	0.732
0.6	0	0.15	0.884	0.648	0.99	0.05	0.15	0.996	0.406
0.6	0.05	0.15	0.78	0.682	0.99	0.05	0.15	0.99	0.566
0.6	0.15	0.15	0.918	0.946	0.99	0.15	0.15	0.9	0.934
0.6	0.15	0.05	0.812	0.692	0.99	0.15	0.05	0.98	0.654
0.6	0.15	0	0.908	0.78	0.99	0.15	0.05	0.996	0.764

**Table 4:** Empirical powers for tests JT and CP with 4 ordinal categories(*k* = 3).

*ρ*	*α*_1_	*β*_1_	JT	CP	*ρ*	*α*_1_	*β*_1_	JT	CP
*α*_0_ = 0.2, *β*_0_ = 0.2									
0.3	0	0.15	0.846	0.784	0.7	0	0.15	0.968	0.692
0.3	0.05	0.15	0.83	0.81	0.7	0.05	0.15	0.868	0.7
0.3	0.15	0.15	0.96	0.978	0.7	0.15	0.15	0.91	0.946
0.3	0.15	0.05	0.842	0.872	0.7	0.15	0.05	0.934	0.79
0.3	0.15	0	0.872	0.856	0.7	0.15	0	0.992	0.816
0.4	0	0.15	0.868	0.776	0.8	0	0.15	0.994	0.672
0.4	0.05	0.15	0.824	0.804	0.8	0.05	0.15	0.956	0.76
0.4	0.15	0.15	0.946	0.968	0.8	0.15	0.15	0.89	0.946
0.4	0.15	0.05	0.878	0.85	0.8	0.15	0.05	0.956	0.76
0.4	0.15	0	0.906	0.858	0.8	0.15	0	0.996	0.806
0.5	0	0.15	0.878	0.716	0.9	0	0.15	0.996	0.636
0.5	0.05	0.15	0.798	0.732	0.9	0.05	0.15	0.978	0.642
0.5	0.15	0.15	0.942	0.964	0.9	0.15	0.15	0.89	0.93
0.5	0.15	0.05	0.868	0.822	0.9	0.15	0.05	0.998	0.712
0.5	0.15	0	0.918	0.822	0.9	0.15	0	0.998	0.766
0.6	0	0.15	0.946	0.738	0.99	0	0.15	0.998	0.626
0.6	0.05	0.15	0.83	0.772	0.99	0.05	0.15	0.996	0.644
0.6	0.15	0.15	0.916	0.938	0.99	0.15	0.15	0.852	0.874
0.6	0.15	0.05	0.9	0.824	0.99	0.15	0.05	0.996	0.71
0.6	0.15	0	0.962	0.84	0.99	0.15	0	0.998	0.756
*α*_0_ = 1.2, *β*_0_ = 1.2									
0.3	0	0.15	0.696	0.618	0.7	0	0.15	0.856	0.552
0.3	0.05	0.15	0.706	0.688	0.7	0.05	0.15	0.722	0.584
0.3	0.15	0.15	0.936	0.968	0.7	0.15	0.15	0.9	0.93
0.3	0.15	0.05	0.816	0.874	0.7	0.15	0.05	0.918	0.81
0.3	0.15	0	0.836	0.872	0.7	0.15	0	0.972	0.85
0.4	0	0.15	0.72	0.644	0.8	0	0.15	0.948	0.548
0.4	0.05	0.15	0.698	0.69	0.8	0.05	0.15	0.774	0.608
0.4	0.15	0.15	0.936	0.946	0.8	0.15	0.15	0.888	0.93
0.4	0.15	0.05	0.844	0.862	0.8	0.15	0.05	0.93	0.796
0.4	0.15	0	0.882	0.846	0.8	0.15	0	0.988	0.818
0.5	0	0.15	0.744	0.586	0.9	0	0.15	0.988	0.482
0.5	0.05	0.15	0.682	0.644	0.9	0.05	0.15	0.876	0.552
0.5	0.15	0.15	0.912	0.946	0.9	0.15	0.15	0.87	0.916
0.5	0.15	0.05	0.866	0.834	0.9	0.15	0.05	0.98	0.744
0.5	0.15	0	0.9	0.818	0.9	0.15	0	0.996	0.798
0.6	0	0.15	0.784	0.594	0.99	0	0.15	0.996	0.484
0.6	0.05	0.15	0.694	0.66	0.99	0.05	0.15	0.998	0.548
0.6	0.15	0.15	0.904	0.928	0.99	0.15	0.15	0.842	0.872
0.6	0.15	0.05	0.89	0.836	0.99	0.15	0.05	0.998	0.756
0.6	0.15	0	0.934	0.85	0.99	0.15	0	0.994	0.776

For the first scenario with *k* = 2, the final ordinal outcome *Y*_2_ can be obtained by dichotomizing *Z* into 1, 2, 3 with the thresholds of *γ*_1_ = 0 and *γ*_2_ = 1. The nominal significant level is set to be 0.05 to calculate the empirical type I error rate and power. All results are calculated based on 500 replicates. From Table 1, we can see that both JT and CP have correct type I error rate since the values are always close to the nominal significance level of 0.05. For example, when *α*_0_ = 0.2, *β*_0_ = 0.2, and *ρ* = 0.3, the empirical type I error rates of JT and CP are 0.047 and 0.052, respectively. For example, when *α*_0_ = 1.2, *β*_0_ = 1.2, and *ρ* = 0.9, the empirical type I error rates of JT and CP are 0.048 and 0.053, respectively. From Table 3, it can be seen that the performance of CP is unsatisfactory when the correlation coefficient *ρ* is large. However, our proposed test JT always has more desirable powers than CP when parameter *α*_1_ is much smaller than *β*_1_ and the correlation between *Y*_1_ and *Y*_2_ is not weak. For example, when *α*_0_ = 0.2, *β*_0_ = 0.2, *α*_1_ = 0.05, *β*_1_ = 0.15, and *ρ* = 0.9, the powers of of JT and CP are 0.922 and 0.57, respectively. When *α*_0_ = 1.2, *β*_0_ = 1.2, *α*_1_ = 0.05, *β*_1_ = 0.15, and *ρ* = 0.8, the powers of of JT and CP are 0.876 and 0.674, respectively. Another example is that when *α*_0_ = 0.2, *β*_0_ = 0.2, *α*_1_ = 0.05, *β*_1_ = 0.15, and *ρ* = 0.7, the powers of of JT and CP are 0.756 and 0.582, respectively. In addition to this, when *α*_0_ = 1.2, *β*_0_ = 1.2, *α*_1_ = 0.05, *β*_1_ = 0.15, and *ρ* = 0.5, the powers of of JT and CP are 0.716 and 0.658, respectively. When the parameters *α*_1_ is larger than or equal to *β*_1_, the powers of the proposed test JT and CP are nearly the same. For example, when *α*_0_ = 0.2, *β*_0_ = 0.2,*α*_1_ = 0.15, *β*_1_ = 0, and *ρ* = 0.3, the powers of of JT and CP are 0.8 and 0.792, respectively. The powers of of JT and CP are 0.832 and 0.866 respectively, when *α*_0_ = 1.2, *β*_0_ = 1.2, *α*_1_ = 0.15, *β*_1_ = 0.15, and *ρ* = 0.9.

For the second scenario with *k* = 3, the final ordinal outcome *Y*_2_ can be obtained by dichotomizing *Z* into 1, 2, 3, 4 with the thresholds of *γ*_1_ = −0.6, *γ*_2_ = 0 and *γ*_3_ = 0.6. The nominal significant level is set to be 0.05 to calculate the empirical type I error rate and power. All results are calculated based on 500 replicates. From Table 2, we can see that both JT and CP have correct type I error rate since the values are always close to the nominal significance level of 0.05. For example, when *α*_0_ = 0.2, *β*_0_ = 0.2, and *ρ* = 0.4, the empirical type I error rates of JT and CP are 0.05 and 0.046, respectively. For example, when *α*_0_ = 1.2, *β*_0_ = 1.2, and *ρ* = 0.8, the empirical type I error rates of JT and CP are 0.046 and 0.049, respectively. From Table 3, it can be seen that the performance of CP is unsatisfactory when the correlation coefficient *ρ* is large. However, our proposed test JT always has more desirable powers than CP when parameter *α*_1_ is much smaller than *β*_1_ and the correlation between *Y*_1_ and *Y*_2_ is not weak. For example, when *α*_0_ = 0.2, *β*_0_ = 0.2, *α*_1_ = 0, *β*_1_ = 0.15, and *ρ* = 0.9, the powers of of JT and CP are 0.996 and 0.636, respectively. When *α*_0_ = 1.2, *β*_0_ = 1.2, *α*_1_ = 0.05, *β*_1_ = 0.15, and *ρ* = 0.8, the powers of of JT and CP are 0.774 and 0.608, respectively. Another example is that when *α*_0_ = 0.2, *β*_0_ = 0.2, *α*_1_ = 0, *β*_1_ = 0.15, and *ρ* = 0.7, the powers of of JT and CP are 0.968 and 0.692, respectively. In addition to this, when *α*_0_ = 1.2, *β*_0_ = 1.2, *α*_1_ = 0, *β*_1_ = 0.15, and *ρ* = 0.5, the powers of of JT and CP are 0.744 and 0.586, respectively. When the parameters *α*_1_ is larger than or equal to *β*_1_, the powers of the proposed test JT and CP are nearly the same. For example, when *α*_0_ = 0.2, *β*_0_ = 0.2,*α*_1_ = 0.15, *β*_1_ = 0, and *ρ* = 0.3, the powers of of JT and CP are 0.872 and 0.856, respectively. The powers of of JT and CP are 0.87 and 0.916 respectively, when *α*_0_ = 1.2, *β*_0_ = 1.2, *α*_1_ = 0.15, *β*_1_ = 0.15, and *ρ* = 0.9.

### Real Data Analysis

To further explore the performance of JT and CP on the testing for the association between multiple outcomes of mixed types and interested covariates, we apply them to the mental health study^1^ in Florida, USA which can be downloaded in www.stat.ufl.edu/aa/glm/data. Mental impairment acts as the ordinal response and life event index which is a composite measure of the number and severity of important life events acts as the continuous response, and socioeconomic status(SES) acts as a 1-dimensional covariate. There are totally 40 samples. The 40 observations analyzed here are merely reflective of patterns found with much larger sample in the study mentioned in introduction section. There are 4 categories about mental impairment values being 1, 2, 3 and 4, which means *k* = 3. Our aim is to test whether the Mental impairment or life event index is associated with SES simultaneously. The p-values of JT and CP for testing this association are 0.042 and 0.46 by R software, which indicates that the proposed method can identify the association under significance level 0.05.

## Discussion

In this paper, we propose a joint model for modeling the association between a bivariate with a continuous outcome and an ordinal outcome by using a latent variable. We conduct some statistical inferences on the parameters in the proposed joint model. Furthermore, a test method is proposed to test whether the the continuous response or ordinal response is associated with covariates. Extensive simulations are conducted to assess the performances of the proposed test procedure. From the simulation results, the proposed method always outperforms the combined p-value method when the correlation of the continuous response and the ordinal response is not weak. Application to a real data analysis further demonstrates the superiority of the new method. When *Y*_1_ and *Y*_2_ act as traits related to genetic, and *G* acts as genotypes, our proposed joint model and test can be applied in modern genome wide association study analysis as Hu *et al*.^17^. In addition, when the dimension number *p* and categories related number *k* become large, it is hard for JT to solve a large number of equations. A feasible approach for ordinal data is to build the test based on ranks^18, 19^. Our analysis is based on an assumption that the observations are from a bivariate normal distribution. When this assumption is not satisfied, a robust method is more appealing. So it deserves further study.

## Methods

### Statistical Inference on Joint Model

In this part, we make statistical inference by the use of MLE statistical theory. Based on the joint model in results section, the likelihood function for unknown parameters can be given by

8 $$\begin{array}{rcl}{L}_{n}({\alpha }_{0}^{\ast },{\beta }_{0}^{\ast \ast },{\alpha }^{\ast },{\beta }^{\ast \ast },{\theta }^{\ast },{\gamma }_{1}^{\ast },\cdots ,{\gamma }_{k}^{\ast },{\sigma }_{1}^{2}) & = & \prod _{i=1}^{n}{s}_{i}({\beta }_{0}^{\ast \ast },{\beta }^{\ast \ast },{\theta }^{\ast },{\gamma }_{1}^{\ast },\cdots ,{\gamma }_{k}^{\ast })\frac{1}{\sqrt{2\pi {\sigma }_{1}^{2}}}{\rm{e}}{\rm{x}}{\rm{p}}\\  &  & -\frac{{({Y}_{1i}-{\alpha }_{0}^{\ast }/{\theta }^{\ast }-{G}_{i}^{\tau }{\alpha }^{\ast }/{\theta }^{\ast })}^{2}}{2{\sigma }_{1}^{2}},\end{array}$$

where

9 $$\begin{array}{rcl}{s}_{i}({\beta }_{0}^{\ast \ast },{\beta }^{\ast \ast },{\gamma }_{1}^{\ast },\cdots ,{\gamma }_{k}^{\ast }) & = & {\rm{\Phi }}({\gamma }_{1}^{\ast }-{\beta }_{0}^{\ast \ast }-{G}_{i}^{\tau }{\beta }^{\ast \ast }-{Y}_{1i}{\theta }^{\ast }){I}_{\{{Y}_{2i}\mathrm{=1\}}}\\  &  & +[1-{\rm{\Phi }}({\gamma }_{k}^{\ast }-{\beta }_{0}^{\ast \ast }-{G}_{i}^{\tau }{\beta }^{\ast \ast }-{Y}_{1i}{\theta }^{\ast })]{I}_{\{{Y}_{2i}=k+\mathrm{1\}}}\\  &  & +\sum _{j=2}^{k}[{\rm{\Phi }}({\gamma }_{j}^{\ast }-{\beta }_{0}^{\ast \ast }-{G}_{i}^{\tau }{\beta }^{\ast \ast }-{Y}_{1i}{\theta }^{\ast })\\  &  & -{\rm{\Phi }}({\gamma }_{j-1}^{\ast }-{\beta }_{0}^{\ast \ast }-{G}_{i}^{\tau }{\beta }^{\ast \ast }-{Y}_{1i}{\theta }^{\ast })]{I}_{\{{Y}_{2i}=j\}}.\end{array}$$

The log-likelihood function is

10 $$\begin{array}{rcl}{l}_{n}({\alpha }_{0}^{\ast },{\beta }_{0}^{\ast \ast },{\alpha }^{\ast },{\beta }^{\ast \ast },{\theta }^{\ast },{\gamma }_{1}^{\ast },\cdots ,{\gamma }_{k}^{\ast },{\sigma }_{1}^{2}) & = & \sum _{{Y}_{2i}=1}\mathrm{log}[{\rm{\Phi }}({\gamma }_{1}^{\ast }-{\beta }_{0}^{\ast \ast }-{G}_{i}^{\tau }{\beta }^{\ast \ast }-{Y}_{1i}{\theta }^{\ast })]\\  &  & +\sum _{{Y}_{2i}=k+1}\mathrm{log}[1-{\rm{\Phi }}({\gamma }_{k}^{\ast }-{\beta }_{0}^{\ast \ast }-{G}_{i}^{\tau }{\beta }^{\ast \ast }-{Y}_{1i}{\theta }^{\ast })]\\  &  & +\sum _{j=2}^{k}\sum _{{Y}_{2i}=j}\mathrm{log}\,[{\rm{\Phi }}({\gamma }_{j}^{\ast }-{\beta }_{0}^{\ast \ast }-{G}_{i}^{\tau }{\beta }^{\ast \ast }-{Y}_{1i}{\theta }^{\ast })\\  &  & -{\rm{\Phi }}({\gamma }_{j-1}^{\ast }-{\beta }_{0}^{\ast \ast }-{G}_{i}^{\tau }{\beta }^{\ast \ast }-{Y}_{1i}{\theta }^{\ast })]\\  &  & -\sum _{i=1}^{n}[\frac{{({Y}_{1i}-\frac{{\alpha }_{0}^{\ast }}{{\theta }^{\ast }}-{G}_{i}^{\tau }\frac{{\alpha }^{\ast }}{{\theta }^{\ast }})}^{2}}{2{\sigma }_{1}^{2}}+\frac{\mathrm{log}({\sigma }_{1}^{2})}{2}+\frac{\mathrm{log}(2\pi )}{2}].\end{array}$$

The MLEs of parameters ${\alpha }_{0}^{\ast }$, ${\beta }_{0}^{\ast \ast }$, *α* ^*^, *β* ^**^, *θ* ^*^, ${\gamma }_{1}^{\ast },\cdots ,{\gamma }_{k}^{\ast }$, and ${\sigma }_{1}^{2}$ can be obtained by maximizing the log-likelihood function which is implemented by solve the following score equations group

11 $$\{\begin{array}{cc}\frac{\partial }{\partial {\alpha }_{0}^{\ast }}{l}_{n}({\alpha }_{0}^{\ast },{\beta }_{0}^{\ast \ast },{\alpha }^{\ast },{\beta }^{\ast \ast },{\theta }^{\ast },{\gamma }_{1}^{\ast },\cdots ,{\gamma }_{k}^{\ast },{\sigma }_{1}^{2}) & \mathrm{=0}\\ \frac{\partial }{\partial {\beta }_{0}^{\ast \ast }}{l}_{n}({\alpha }_{0}^{\ast },{\beta }_{0}^{\ast \ast },{\alpha }^{\ast },{\beta }^{\ast \ast },{\theta }^{\ast },{\gamma }_{1}^{\ast },\cdots ,{\gamma }_{k}^{\ast },{\sigma }_{1}^{2}) & \mathrm{=0}\\ \frac{\partial }{\partial {\alpha }^{\ast }}{l}_{n}({\alpha }_{0}^{\ast },{\beta }_{0}^{\ast \ast },{\alpha }^{\ast },{\beta }^{\ast \ast },{\theta }^{\ast },{\gamma }_{1}^{\ast },\cdots ,{\gamma }_{k}^{\ast },{\sigma }_{1}^{2}) & \mathrm{=0}\\ \frac{\partial }{\partial {\beta }^{\ast \ast }}{l}_{n}({\alpha }_{0}^{\ast },{\beta }_{0}^{\ast \ast },{\alpha }^{\ast },{\beta }^{\ast \ast },{\theta }^{\ast },{\gamma }_{1}^{\ast },\cdots ,{\gamma }_{k}^{\ast },{\sigma }_{1}^{2}) & \mathrm{=0}\\ \frac{\partial }{\partial {\theta }^{\ast }}{l}_{n}({\alpha }_{0}^{\ast },{\beta }_{0}^{\ast \ast },{\alpha }^{\ast },{\beta }^{\ast \ast },{\theta }^{\ast },{\gamma }_{1}^{\ast },\cdots ,{\gamma }_{k}^{\ast },{\sigma }_{1}^{2}) & \mathrm{=0}\\ \frac{\partial }{\partial {\gamma }_{1}^{\ast }}{l}_{n}({\alpha }_{0}^{\ast },{\beta }_{0}^{\ast \ast },{\alpha }^{\ast },{\beta }^{\ast \ast },{\theta }^{\ast },{\gamma }_{1}^{\ast },\cdots ,{\gamma }_{k}^{\ast },{\sigma }_{1}^{2}) & \mathrm{=0}\\ \vdots  & \vdots \\ \frac{\partial }{\partial {\gamma }_{k}^{\ast }}{l}_{n}({\alpha }_{0}^{\ast },{\beta }_{0}^{\ast \ast },{\alpha }^{\ast },{\beta }^{\ast \ast },{\theta }^{\ast },{\gamma }_{1}^{\ast },\cdots ,{\gamma }_{k}^{\ast },{\sigma }_{1}^{2}) & \mathrm{=0}\\ \frac{\partial }{\partial ({\sigma }_{1}^{2})}{l}_{n}({\alpha }_{0}^{\ast },{\beta }_{0}^{\ast \ast },{\alpha }^{\ast },{\beta }^{\ast \ast },{\theta }^{\ast },{\gamma }_{1}^{\ast },\cdots ,{\gamma }_{k}^{\ast },{\sigma }_{1}^{2}) & \mathrm{=0}\end{array}$$

Denote ${\mu }_{i}={\beta }_{0}^{\ast \ast }+{G}_{i}^{\tau }{\beta }^{\ast \ast }+{Y}_{1i}{\theta }^{\ast }$. With $a={({\alpha }_{0}^{\ast },{\beta }_{0}^{\ast \ast },{\alpha }^{\ast },{\beta }^{\ast \ast },{\theta }^{\ast },{\gamma }_{1}^{\ast },\cdots ,{\gamma }_{k}^{\ast },{\sigma }_{1}^{2})}^{\tau }$ in results section, we can write ${l}_{n}({\alpha }_{0}^{\ast },{\beta }_{0}^{\ast \ast },{\alpha }^{\ast },{\beta }^{\ast },{\theta }^{\ast },{\gamma }_{1}^{\ast },\cdots ,{\gamma }_{k}^{\ast },{\sigma }_{1}^{2})$ in a simple form as follows:

12 $$\begin{array}{rcl}{l}_{n}(a) & = & \sum _{{Y}_{2i}=1}\mathrm{log}[{\rm{\Phi }}({\gamma }_{1}^{\ast }-{\mu }_{i})]+\sum _{{Y}_{2i}=k+1}\mathrm{log}[1-{\rm{\Phi }}({\gamma }_{k}^{\ast }-{\mu }_{i})]\\  &  & +\sum _{j=2}^{k}\sum _{{Y}_{2i}=j}\mathrm{log}\,[{\rm{\Phi }}({\gamma }_{j}^{\ast }-{\mu }_{i})-{\rm{\Phi }}({\gamma }_{j-1}^{\ast }-{\mu }_{i})]\\  &  & -\sum _{i=1}^{n}[\frac{{({Y}_{1i}-\frac{{\alpha }_{0}^{\ast }}{{\theta }^{\ast }}-{G}_{i}^{\tau }\frac{{\alpha }^{\ast }}{{\theta }^{\ast }})}^{2}}{2{\sigma }_{1}^{2}}+\frac{\mathrm{log}({\sigma }_{1}^{2})}{2}+\frac{\mathrm{log}(2\pi )}{2}].\end{array}$$

The detailed expressions of the score functions(left sides of (11)) are provided as follows:

13 $$\frac{\partial }{\partial {\alpha }_{0}^{\ast }}{l}_{n}(a)=\sum _{i=1}^{n}[\frac{({Y}_{1i}-\frac{{\alpha }_{0}^{\ast }}{{\theta }^{\ast }}-\frac{{G}_{i}^{\tau }{\alpha }^{\ast }}{{\theta }^{\ast }})}{2{\sigma }_{1}^{2}}],$$

14 $$\frac{\partial }{\partial {\beta }_{0}^{\ast \ast }}{l}_{n}(a)=-\sum _{{Y}_{2i}=1}\frac{\varphi ({\gamma }_{1}^{\ast }-{\mu }_{i})}{{\rm{\Phi }}({\gamma }_{1}^{\ast }-{\mu }_{i})}-\sum _{{Y}_{2i}=k+1}\frac{-\varphi ({\gamma }_{k}^{\ast }-{\mu }_{i})}{1-{\rm{\Phi }}({\gamma }_{k}^{\ast }-{\mu }_{i})}-\sum _{j\mathrm{=2}}^{k}\sum _{{Y}_{2i}=j}\frac{\varphi ({\gamma }_{j}^{\ast }-{\mu }_{i})-\varphi ({\gamma }_{j-1}^{\ast }-{\mu }_{i})}{{\rm{\Phi }}({\gamma }_{j}^{\ast }-{\mu }_{i})-{\rm{\Phi }}({\gamma }_{j-1}^{\ast }-{\mu }_{i})},$$

15 $$\frac{\partial }{\partial {\alpha }^{\ast }}{l}_{n}(a)=\sum _{i=1}^{n}[\frac{({Y}_{1i}-\frac{{\alpha }_{0}^{\ast }}{{\theta }^{\ast }}-\frac{{G}_{i}^{\tau }{\alpha }^{\ast }}{{\theta }^{\ast }})}{2{\sigma }_{1}^{2}}{G}_{i}],$$

16 $$\frac{\partial }{\partial {\beta }^{\ast \ast }}{l}_{n}(a)=-\sum _{{Y}_{2i}\mathrm{=1}}\frac{\varphi ({\gamma }_{1}^{\ast }-{\mu }_{i})}{{\rm{\Phi }}({\gamma }_{1}^{\ast }-{\mu }_{i})}{G}_{i}-\sum _{{Y}_{2i}=k+1}\frac{-\varphi ({\gamma }_{k}^{\ast }-{\mu }_{i})}{1-{\rm{\Phi }}({\gamma }_{k}^{\ast }-{\mu }_{i})}{G}_{i}-\sum _{j=2}^{k}\sum _{{Y}_{2i}=j}\frac{\varphi ({\gamma }_{j}^{\ast }-{\mu }_{i})-\varphi ({\gamma }_{j-1}^{\ast }-{\mu }_{i})}{{\rm{\Phi }}({\gamma }_{j}^{\ast }-{\mu }_{i})-{\rm{\Phi }}({\gamma }_{j-1}^{\ast }-{\mu }_{i})}{G}_{i},$$

17 $$\begin{array}{rcl}\frac{\partial }{\partial {\theta }^{\ast }}{l}_{n}(a) & = & -\sum _{{Y}_{2i}=1}\frac{\varphi ({\gamma }_{1}^{\ast }-{\mu }_{i})}{{\rm{\Phi }}({\gamma }_{1}^{\ast }-{\mu }_{i})}{Y}_{1i}-\sum _{{Y}_{2i}=k+1}\frac{-\varphi ({\gamma }_{k}^{\ast }-{\mu }_{i})}{1-{\rm{\Phi }}({\gamma }_{k}^{\ast }-{\mu }_{i})}{Y}_{1i}\\  &  & -\sum _{j=2}^{k}\sum _{{Y}_{2i}=j}\frac{\varphi ({\gamma }_{j}^{\ast }-{\mu }_{i})-\varphi ({\gamma }_{j-1}^{\ast }-{\mu }_{i})}{{\rm{\Phi }}({\gamma }_{j}^{\ast }-{\mu }_{i})-{\rm{\Phi }}({\gamma }_{j-1}^{\ast }-{\mu }_{i})}{Y}_{1i}\\  &  & -\sum _{i=1}^{n}[\frac{({Y}_{1i}-\frac{{G}_{i}^{\tau }{\beta }^{\ast }}{{\theta }^{\ast }}){G}_{i}^{\tau }{\beta }^{\ast }}{2{\sigma }_{1}^{2}{({\beta }^{\ast })}^{2}}],\end{array}$$

18 $$\frac{\partial }{\partial {\gamma }_{1}^{\ast }}{l}_{n}(a)=\sum _{{Y}_{2i}=1}\frac{\varphi ({\gamma }_{1}^{\ast }-{\mu }_{i})}{{\rm{\Phi }}({\gamma }_{1}^{\ast }-{\mu }_{i})}+\sum _{{Y}_{2i}=2}\frac{-\varphi ({\gamma }_{1}^{\ast }-{\mu }_{i})}{{\rm{\Phi }}({\gamma }_{2}^{\ast }-{\mu }_{i})-{\rm{\Phi }}({\gamma }_{1}^{\ast }-{\mu }_{i})},$$

19 $$\begin{array}{rcl}\frac{\partial }{\partial {\gamma }_{j}^{\ast }}{l}_{n}(a) & = & \sum _{{Y}_{2i}=j}\frac{\varphi ({\gamma }_{j}^{\ast }-{\mu }_{i})}{{\rm{\Phi }}({\gamma }_{j}^{\ast }-{\mu }_{i})-{\rm{\Phi }}({\gamma }_{j-1}^{\ast }-{\mu }_{i})}\\  &  & +\sum _{{Y}_{2i}=j+1}\frac{-\varphi ({\gamma }_{j}^{\ast }-{\mu }_{i})}{{\rm{\Phi }}({\gamma }_{j+1}^{\ast }-{\mu }_{i})-{\rm{\Phi }}({\gamma }_{j}^{\ast }-{\mu }_{i})},\,{\rm{for}}\,j=\mathrm{2,}\cdots ,k-\mathrm{1,}\end{array}$$

20 $$\frac{\partial }{\partial {\gamma }_{k}^{\ast }}{l}_{n}(a)=\sum _{{Y}_{2i}=k}\frac{\varphi ({\gamma }_{k}^{\ast }-{\mu }_{i})}{{\rm{\Phi }}({\gamma }_{k}^{\ast }-{\mu }_{i})-{\rm{\Phi }}({\gamma }_{k-1}^{\ast }-{\mu }_{i})}+\sum _{{Y}_{2i}=k+1}\frac{-\varphi ({\gamma }_{k}^{\ast }-{\mu }_{i})}{1-{\rm{\Phi }}({\gamma }_{k}^{\ast }-{\mu }_{i})},$$

21 $$\frac{\partial }{\partial {\sigma }_{1}^{2}}{l}_{n}(a)=\sum _{i=1}^{n}[\frac{{({Y}_{1i}--\frac{{\alpha }_{0}^{\ast }}{{\theta }^{\ast }}-\frac{{G}_{i}^{\tau }{\alpha }^{\ast }}{{\theta }^{\ast }})}^{2}}{2{\sigma }_{1}^{2}}-\frac{1}{2{\sigma }_{1}^{2}}].$$

We can obtain MLE $\hat{a}={(\widehat{{\alpha }_{0}^{\ast }},\widehat{{\beta }_{0}^{\ast \ast }},\widehat{{\alpha }^{\ast }},\widehat{{\beta }^{\ast \ast }},\widehat{{\theta }^{\ast }},\widehat{{\gamma }_{1}^{\ast }},\cdots ,\widehat{{\gamma }_{k}^{\ast }},\widehat{{\sigma }_{1}^{2}})}^{\tau }$ for $a=({\alpha }_{0}^{\ast },{\beta }_{0}^{\ast \ast },{\alpha }^{\ast },{\beta }^{\ast \ast },{\theta }^{\ast },$ ${\gamma }_{1}^{\ast },\cdots ,{\gamma }_{k}^{\ast },{\sigma }_{1}^{2}{)}^{\tau }$ by solving the equations group (11) based on the Newton’s method in R software. According to the statistical asymptotisc theory^14^, under the null hypothesis where neither the continuous response *Y*_1_ nor the ordinal response *Y*_2_ is associated with covariates *G*, we have $\sqrt{n}({\hat{a}}^{\tau }-{a}^{\tau })$ asymptotically follows from the normal distribution with mean vector 0_(2*p*+*k*+4)×1_ and covariance *I* ^−1^(*a*), where *I*(*a*) is the Fisher information matrix taking the following form

22 $$I(a)=-{(\begin{array}{ccccc}E[\frac{{\partial }^{2}ln(a)}{{(\partial {\alpha }_{0}^{\ast })}^{2}}] & E[\frac{{\partial }^{2}ln(a)}{\partial {\alpha }_{0}^{\ast }\partial {\beta }_{0}^{\ast \ast }}] & E[\frac{{\partial }^{2}ln(a)}{\partial {\alpha }_{0}^{\ast }\partial {\alpha }_{1}^{\ast }}] & \cdots  & E[\frac{{\partial }^{2}ln(a)}{\partial {\alpha }_{0}^{\ast }\partial ({\sigma }_{1}^{2})}]\\ E[\frac{{\partial }^{2}ln(a)}{\partial {\beta }_{0}^{\ast }\partial {\alpha }_{0}^{\ast }}] & E[\frac{{\partial }^{2}ln(a)}{{(\partial {\beta }_{0}^{\ast })}^{2}}] & E[\frac{{\partial }^{2}ln(a)}{\partial {\beta }_{0}^{\ast }\partial {\alpha }_{1}^{\ast }}] & \cdots  & E[\frac{{\partial }^{2}ln(a)}{\partial {\alpha }_{1}^{\ast }\partial ({\sigma }_{1}^{2})}]\\ \vdots  & \vdots  & \vdots  & \ddots  & \vdots \\ E[\frac{{\partial }^{2}ln(a)}{\partial ({\sigma }_{1}^{2})\partial {\alpha }_{0}^{\ast }}] & E[\frac{{\partial }^{2}ln(a)}{\partial ({\sigma }_{1}^{2})\partial {\beta }_{0}^{\ast }}] & E[\frac{{\partial }^{2}ln(a)}{\partial ({\sigma }_{1}^{2})\partial {\alpha }_{1}^{\ast }}] & \cdots  & E[\frac{{\partial }^{2}ln(a)}{{(\partial ({\sigma }_{1}^{2}))}^{2}}]\end{array})}_{\mathrm{(2}p+k+\mathrm{4)}\times \mathrm{(2}p+k+\mathrm{4)}}.$$

### Derivation of Covariance Matrix *W*

Denote *V* as sub-matrix in *I* ^−1^(*a*) with the 3rd to the (2*p* + 2)th rows and columns, and $$H={(\begin{array}{c}{I}_{p}{\mathrm{,0}}_{p}\\ {I}_{p},{I}_{p}\end{array})}_{2p\times 2p}$$, where *I*_*p*_ is the identity matrix with dimension *p*, 0_*p*_ is the null matrix with all elements 0 and dimension *p*. According to asymptotic theorems^14^, as sample size *n* goes for infinity, $${(\begin{array}{c}\widehat{{\alpha }^{\ast }}\\ \widehat{{\beta }^{\ast \ast }}\end{array})}_{2p\times 1}\mathop{\longrightarrow }\limits^{D}N((\begin{array}{c}{\alpha }^{\ast }\\ {\beta }^{\ast \ast }\end{array}),V).$$ By some algebra, we have $$\widehat{\eta }=H(\begin{array}{c}\widehat{{\alpha }^{\ast }}\\ \widehat{{\beta }^{\ast \ast }}\end{array})$$. We can obtain that $\widehat{\eta }$ is an asymptotic unbiased estimate for parameters *η*; and $\widehat{\eta }-\eta \,\mathop{\longrightarrow }\limits^{D}N(\mathrm{0,}\,HV{H}^{\tau })$ as sample size *n* goes for infinity based on the multi-normal distribution theory^15^.
